# Cord blood-derived biologics lead to robust axonal regeneration in benzalkonium chloride-injured mouse corneas by modulating the Il-17 pathway and neuropeptide Y

**DOI:** 10.1186/s10020-023-00772-w

**Published:** 2024-01-03

**Authors:** Ruojing Huang, Caiying Su, Na Zhang, Congying Shi, Guangming Pu, Yong Ding, Wei Wei, Jiansu Chen

**Affiliations:** 1 The First Affiliated Hospital of Jinan University, Tianhe District, Guangzhou, 510630 Guangdong Province China; 2 Peking University Shenzhen Hospital, Futian District, Shenzhen, 518036 Guangdong Province China; 3 School of Chemical and Biomedical Engineering, Nanyang Technological University, 62 Nanyang Dr, Singapore, 637459 Singapore; 4 Institution of Guangdong Cord Blood Bank, Guangdong Women and Children Hospital, Guangzhou, 510705 Guangdong Province China; 5 Department of Experimental Center, Guangzhou Municipality Tianhe Nuoya Bio-Engineering Co., Ltd, Guangzhou, 510705 Guangdong Province China; 6 Jinan University Affiliated Heyuan Hospital, Guangzhou, 517000 Guangdong Province China; 7 Institute of Ophthalmology, Medical College, Jinan University, Guangzhou, 510630 Guangdong Province China

**Keywords:** Umbilical cord blood, Serum, Plasma-rich platelet, Inflammation, Nerve regeneration, Cornea, Benzalkonium chloride

## Abstract

**Background:**

Umbilical cord blood-derived therapeutics, such as serum (UCS) and platelet-rich plasma (UCPRP), are popular treatment options in clinical trials and can potentially be utilized to address a clinically unmet need caused by preservatives, specifically benzalkonium chloride (BAK), present in ophthalmic formulations. As current clinical interventions for secondary injuries caused by BAK are suboptimal, this study will explore the feasibility of utilizing UCS and UCPRP for cornea treatment and investigate the underlying mechanisms associated with this approach.

**Methods:**

Mice’s corneas were administered BAK to induce damage. UCS and UCPRP were then utilized to attempt to treat the injuries. Ocular tests were performed on the animals to evaluate recovery, while immunostaining, RNA-seq, and subsequent bioinformatics analysis were conducted to investigate the treatment mechanism.

**Results:**

BAK administration led to widespread inflammatory responses in the cornea. Subsequent treatment with UCS and UCPRP led to the downregulation of immune-related ‘interactions between cytokine receptors’ and ‘IL-17 signaling’ pathways. Although axonal enhancers such as Ngf, Rac2, Robo2, Srgap1, and Rock2 were found to be present in the injured group, robust axonal regeneration was observed only in the UCS and UCPRP treatment groups. Further analysis revealed that, as compared to normal corneas, inflammation was not restored to pre-injury levels post-treatment. Importantly, Neuropeptide Y (Npy) was also involved in regulating immune responses, indicating neuroimmune axis interactions.

**Conclusions:**

Cord blood-derived therapeutics are feasible options for overcoming the sustained injuries induced by BAK in the cornea. They also have potential applications in areas where axonal regeneration is required.

**Supplementary Information:**

The online version contains supplementary material available at 10.1186/s10020-023-00772-w.

## Introduction

Autologous blood-derived therapeutics, such as serum and platelet-rich plasma (PRP), are potent and popular treatment options in clinical trials (Alves and Grimalt 2017). Recently, this technology has advanced and extended to umbilical cord blood as a derivative source as well. Compared to autologous blood, umbilical cord blood-derived serum (UCS) and platelet-rich plasma (UCPRP) contain higher levels of growth factors, are readily available, and are more sterile (Yoon et al. 2007; Nadelmann et al. 2021; Rhéaume et al. 2022; Roura et al. 2015). Importantly, they have been utilized in various disease treatments (Wang et al. 2021a; Mazzotta et al. 2022), among which eye disease is one of the most prominent ones. Specifically, Oh et al. observed diminished epithelial defect parameters, improved epithelial integrity, and reduced stromal inflammation and edema in the group treated with UCS versus PBS. This was evident in a mouse model of ocular surface burn induced by NaOH (Oh et al. 2012). Furthermore, Han et al. illustrated significantly reduced epithelial defect areas, diminished opacity levels, and lower expression levels of Tnfa, Il6, MMP-8, and MMP-9 mRNA, as well as a notable decrease in the infiltration of inflammatory cells in the UCS-treated group as compared to the PBS group in a mouse corneal alkali burn model (Han et al. 2019). Finally, Giannaccare et al. demonstrated that the use of cord blood serum eye drops led to a significant improvement in corneal nerve morphology, characterized by increased nerve density and reduced nerve tortuosity, among dry eye (DE) patients (Giannaccare et al. 2017). Despite less than two decades of animal research, their emerging usage in recent clinical trials across multidisciplinary fields has also highlighted their efficacy in achieving the intended treatment outcomes (Miguel-Gómez et al. 2021; Rani et al. 2022). Accordingly, these biologics can potentially be utilized to address a clinically unmet need caused by preservatives present in ophthalmic formulations.

Preservatives in ophthalmic formulations are imperative for maintaining sterility, which in turn prolongs their shelf life and reusability. Although any compounds containing a nonspecific antimicrobial spectrum extending far beyond the test organisms qualify as a preservative, benzalkonium chloride (BAK) remains the most commonly used to date (Tu et al. 2013; Baudouin et al. 2010). As an antimicrobial agent, it primarily functions to disrupt cell membranes and is extremely effective against both Gram-positive and Gram-negative bacteria, as well as fungi (Baudouin et al. 2010). BAK can also serve as a corneal penetration enhancer, allowing the active ingredients in medications to exhibit stronger ocular permeability (Rathore and Majumdar 2006). However, studies have shown that not only do enhanced pharmacokinetic characteristics not necessarily correlate to its efficacy, but the perceived penetrability is a toxic side effect due to major histological changes in the corneal barrier (Jong et al. 1994). Such breaching of corneal integrity impedes treatment since it induces adverse ocular events and may even pose complications in deeper retinal and optic nerve tissues for patients undergoing long-term therapy. Yet despite documentation about its side effects since the 40 s, there remain no better replacements. Preservatives-free formulations serve as an alternative option but non-sterile incidents can lead to repercussions as evidenced by recent clinical cases and the recalls of such eyedrops (Swan 1944; Morelli et al. 2023; Shoji et al. 2023).

Current clinical interventions for ocular surface diseases caused by BAK include artificial tears and immunosuppressive drugs. However, the outcomes are suboptimal and not all patients are responsive to these treatments. While studies seeking to ameliorate the negative effects of BAK have already been conducted previously, there are, to our knowledge, no reports on the effectiveness of UCS and UCPRP in mitigating severe BAK-induced injuries in the cornea (Wang et al. 2021b; Fukuda et al. 2022). Moreover, mechanistic studies for these biologics are lacking and treatment outcomes were mostly predicted based on the presence of initial growth factors. Therefore, in this study, we will be aiming to address both of these aspects.

Our study first tested the effects of BAK on mice’s corneas. Thereafter, we administered UCS and UCPRP and found that these treatments led to significantly improved corneal recovery. Through transcriptional analysis of the mice’s corneas, our results revealed that both UCS and UCPRP primarily led to the downregulation of innate and adaptive immune responses, specifically by damping interactions between cytokine receptors and IL-17 signaling. Concurrent regulation of key axonal regeneration and/or guidance genes such as *Ngf, Rac2, Robo2, Srgap1, Rock2 and Sema4* as well as *Npy* led to robust axonal regeneration in the cornea. Of note, it was found that the inflammatory responses, though mitigated, were not restored to pre-injury levels and remained upregulated as compared to uninjured corneas. Our findings demonstrated the feasibility of employing cord blood-derived therapeutics for overcoming sustained detrimental effects induced by BAK in the cornea and showed that they can potentially be used in other areas where axonal regeneration is required.

## Materials and methods

### UCS and UCPRP preparation

Umbilical cord serum (UCS) and umbilical cord blood platelet-rich plasma (UCPRP) was obtained from Guangdong Province Umbilical Cord Blood Bank. All activities of the Guangdong UCB Bank are conducted in accordance with Chinese laws and regulations. This study has been approved by the Guangdong UCB Bank Ethics Committee.

UCS: The umbilical cord blood was allowed to clot by letting it sit at room temperature for 2 h. It was then centrifuged at 4 °C for 15 min at 3000 rpm, and the clear serum was carefully separated under sterile conditions. The filtered serum was then used as is or stored at − 20 °C for up to 6 months. Three batches of UCS were utilized for the experiments.

UCPRP: To store umbilical cord blood and prevent platelet activation, a test tube containing 3.2% sodium citrate solution was used. In the first centrifugation step, the blood was centrifuged at 600 rpm for 10 min, resulting in three layers. The top two layers were discarded and the tube was centrifuged again at 1500 rpm for 10 min to concentrate the platelets. Following centrifugation, two-thirds of the volume was discarded and the remaining one-third volume at the bottom containing the enriched platelets was collected in a sterile tube which was used as is. Three batches of UCPRP were utilized for the experiments.

### Animals

The C57BL/6 female used in this experiment were provided by the Guangdong Provincial Experimental Animal Center. Female mice were chosen to minimize the influence of gender differences on the experiment. The mice were 6–8 weeks old and of SPF (Specific Pathogen-Free) grade, with no ocular diseases. They were housed in a 12-h light–dark cycle environment (lighting from 0800 to 2000 h) at a temperature of 22 °C ± 2 °C under normal conditions, with unrestricted access to food and water. All animal experimental procedures complied with the guidelines of the National Institutes of Health (NIH) and were approved by the Laboratory Animal Ethics Committee of Jinan University. Every effort was made to minimize the pain and number of animals used in the experiments. Animal grouping information can be found in Additional file 1: Table S1.

### BAK-induced corneal injury in mice

A mouse model of corneal injury was induced by topical application of 0.2% BAK (Sigma, Catalogue: 12060) eye drops in female C57BL/6 mice. The protocol involves administering 5 μL of BAK eye drops to each eye twice daily (at 0800 h and 2000 h) for a consecutive 14-day period. (Xiao et al. 2012; Zhang et al. 2020)

### In vivo, UCS/UCPRP treatment following BAK-induced injury

After the consecutive 14-day period of BAK administration, mice allocated for UCS or UCPRP treatment were provided 5 μL of 20% UCS or 20% UCPRP eye drops thrice daily (at 0800 h, 1400 h, and 2000 h) for a consecutive 10-day period. Mice allocated for saline treatment will only be provided with saline eye drops thrice daily at the same timing and duration. Control mice did not receive any cornea injuries or treatment.

### Corneal fluorescein sodium staining

Fluorescein, an orange dye, is easily detectable under blue light and serves as a valuable tool for assessing the structural integrity of the cornea (Srinivas and Rao 2023). When applied, any abnormalities on the corneal surface are highlighted by the dye, manifesting as green fluorescence under blue light. Researchers can discern the specific location and potential causes of corneal issues by analyzing the size, location, and shape of the resulting staining (Pellegrini et al. 2019; Chen et al. 2017). Specifically, an intraperitoneal injection of 1.25% tribromoethanol (Maikelin Biochemical Technology Co., Ltd, China) was used to anesthetize the mice. Subsequently, 5 µL of 1% fluorescein sodium (Sigma, Catalogue: F6377) was instilled into the cornea and the mice were manually prompted to blink three times to ensure even coverage. A fluorescence microscope equipped with a cobalt blue filter is used for photography and scoring. The scoring method involves dividing the corneal image into four quadrants (cross-shaped division), with each quadrant being assigned a score on a scale of 0 to 4: 0 = no staining, 1 = mild punctate staining, 2 = moderate punctate staining or mild linear staining, 3 = severe linear staining or mild patchy staining (area less than half of the quadrant), 4 = severe patchy staining (area greater than half of the quadrant). The image scoring was performed by one experimenter and a second blinded observer. Each quadrant was scored, and the values for all quadrants were summed. The average of the two observers’ scores was considered the final fluorescein sodium staining score for the cornea. This assessment was conducted on Day 0, Day 7, and Day 14 for preliminary BAK-induced injury without treatment. For animals undergoing UCS or UCPRP treatment, this assessment was conducted on Day 10 after the 14-day BAK-induced injury.

### Corneal opacity scoring

Images were captured using a fluorescence microscope with white light and corneal opacity was evaluated by another blinded researcher. The scoring method utilized a 5-point scale (0–4) as follows: 0 = clear cornea; no opacity, 1 = corneal haze with clear visibility of the iris, 2 = mild corneal opacity with still visible iris, 3 = severe corneal opacity with faintly visible iris, 4 = corneal opacity with no visibility of the iris. This assessment was conducted on Day 0, Day 7, and Day 14 for preliminary BAK-induced injury without treatment. For animals undergoing UCS or UCPRP treatment, this assessment was conducted on Day 10 after the 14-day BAK-induced injury. The evaluation of corneal opacity was conducted by a researcher who was unaware of the experimental conditions, ensuring an unbiased assessment.

### Tear secretion test

An intraperitoneal injection of 1.25% tribromoethanol was used to anesthetize the mice. A phenol red cotton thread (Tianjin Jingming New Technology Development Co., Ltd, China) was placed on the lower fornix of the eye, approximately one-third of the distance from the outer canthus of the lower eyelid, and left in place for 1 min. The length of the cotton thread was measured in millimetres (mm) under a microscope and readings were taken by two observers, with the average value recorded. This test was conducted on Day 0, Day 7, and Day 14 for preliminary BAK-induced injury without treatment. For animals undergoing UCS or UCPRP treatment, this test was conducted on Day 10 after the 14-day BAK-induced injury.

### Tear film break-up time measurement

After anesthetizing the mouse, 1 μL of 0.1% fluorescein sodium was dripped onto the mouse’s conjunctival sac. The mouse was then manually prompted to blink three times to ensure an even distribution of the fluorescein sodium on the corneal surface. The tear film break-up time (TBUT) was determined by observing the time it takes for the first appearance of a black spot, black line, or obvious break in the tear film using a cobalt blue light with a slit lamp. Each eye was tested three times, and the final result was the average of those values. This measurement was conducted on Day 0, Day 7, and Day 14 for preliminary BAK-induced injury without treatment. For animals undergoing UCS or UCPRP treatment, this measurement was conducted on Day 10 after the 14-day BAK-induced injury.

### Corneal sensitivity measurement

Corneal sensitivity measurement was conducted using the Cochet-Bonnet aesthesiometer. In a non-anesthetized and awake state, the mice were gently held by the examiner and probed in the central area of the cornea with a nylon filament. Starting with a predetermined maximum length of the nylon filament (6.0 cm), the length was decreased by 0.5 cm for each subsequent measurement. The process continued until the mice exhibited a noticeable blink response. If the mice blinked effectively, the nylon filament was extended by 0.5 cm, and the measurement was repeated. The longest length of the nylon filament that elicited an effective blink reflex was recorded as the threshold for corneal sensitivity. The procedure was repeated three times, and the average value was calculated as the final result. This measurement was also conducted on Day 0, Day 7, and Day 14 for preliminary BAK-induced injury without treatment. For animals undergoing UCS or UCPRP treatment, this measurement was conducted on Day 10 after the 14-day BAK-induced injury.

### Collection and storage of mice tears

Mice were anesthetized using intraperitoneal injection of 1.25% tribromoethanol. A micro-sampling pipette was then utilized to deliver 2 μL of 0.1% FBS into the mice’s conjunctival sac and blinking was prompted manually. The tears produced were collected through capillary action by using a 10 μL capillary tube to touch the meniscus (tear film on the lower lid). Tears from mice in all the different groups were collected and stored in a − 80 °C freezer.

### Enzyme-linked immunosorbent assay (ELISA)

The concentration of *Muc5ac* in the mice tears was evaluated by performing ELISA as per the manufacturer’s protocol (J&L Biological, China). Briefly, standard wells were set up by adding 50 μL of different concentrations of standard samples. In the blank well, 50 μL of sample diluent was added while in the experimental wells, 40 μL of sample diluent and 10 μL of the test tear fluid was added. Next, 100 μL of horseradish peroxidase (HRP)-conjugated detection antibody was added to each well and the plate was sealed and incubated at 37 °C for 1 h. After the incubation period, the liquid was discarded, patted dry on absorbent paper, and washed 5 times with the wash buffer provided. Subsequently, 50 μL of both substrate solutions A and B were added into each well and incubated at 37 °C for 15 min. Another 50 μL of stop solution was added before measuring their OD at 450 nm with a microplate reader. An Excel spreadsheet was then used to plot the standard curve for determining the concentration of *Muc5ac*.

### RNA extraction and qPCR

The mice were euthanized by cervical dislocation following anesthesia and their eyeballs were removed immediately. They were then placed on ice and the cornea was separated and washed thoroughly with pre-chilled PBS. Four corneas within the same group were then placed into a 15 mL centrifuge tube containing 1 mL of Trizol lysis reagent (Invitrogen, Catalogue: 15596026) and physically ground with a handheld homogenizer. The homogenized samples were then transferred to a 1.5 mL microcentrifuge tube and let sit for 5 min to allow complete separation of the nucleic acid-protein complexes. 200 μL of chloroform was then added and vigorously mixed for 15 s. The tubes were then left at room temperature for another 3 min before centrifuging at 12,000 rpm for 10 min in a refrigerated centrifuge. Following centrifugation, the upper aqueous phase was transferred to a new 1.5 mL microcentrifuge tube, and an equal volume of absolute ethanol was added and mixed. The following step of purification RNA was performed using the total RNA extraction kit (Tiangen, China). Specifically, the mixture was then transferred to an absorption column (CR3) and centrifuged at 12,000 rpm for 30 s in a refrigerated centrifuge. 500 μL of protein removal solution (RD) was next added to the absorption column and the mixture was centrifuged again at 12,000 rpm for 30 s. Following that, 500 μL of wash buffer (RW) was added to the absorption column and centrifuged at 12,000 rpm for 30 s. The washing step was repeated once and residual liquid was removed by centrifuging the absorption column at 12,000 rpm for another 2 min. 30 μL of RNAse-free ddH_2_O was used for reconstitution. Extracted RNA was converted into cDNA using a reverse transcription kit (ReverTra Ace qPCR RT kit, Toyobo Research Reagents, Japan) and a thermal cycler. qPCR was performed in the following conditions: Pre-denaturation at 95 °C for 1 min, denaturation at 95 °C for 15 s, annealing at 60 °C for 30 s, and extension at 72 °C for 30 s. This process is repeated for a total of 40 cycles. Primer information can be found in Additional file 1: Table S2.

### Library construction, QC, and sequencing

Total RNA samples were subjected to oligo(dT) magnetic bead enrichment to isolate mRNA. They were then fragmented into RNA fragments by adding fragmentation reagents and incubating at an appropriate temperature for a specific period. A first-strand synthesis reaction was performed by adding a reverse transcription reaction mixture and incubating it in a PCR machine to synthesize first-strand cDNA. Then, a second-strand synthesis reaction mixture was added, and incubated at an appropriate temperature for a specific period to synthesize double-stranded cDNA. Subsequently, the synthesized double-stranded cDNA was subjected to end repair, followed by the addition of an adenine base to the 3′ ends. The adapter ligation reaction mixture was prepared, incubated at an appropriate temperature for a specific period, and then used to ligate the cDNA with adapters. Next, a PCR reaction mixture was prepared, and a PCR program was set up for the amplification of the ligated products, which were then purified thereafter. The PCR products were next subjected to size selection to remove linear DNA molecules that were not circularized, completing the library construction. The size and concentration of the constructed libraries were assessed using the Agilent 2100 Bioanalyzer and the qualified libraries were subjected to sequencing using the Illumina HiSeq platform. Information regarding RNA quality can be found in Additional file 1: Table S3.

### Analysis of RNA-sequencing results

RNA-seq results were analyzed and plotted using R software with DESeq2 package (Love et al. 2014), SBGNview package (Dong et al. 2022), and their required dependency packages. DEseq2 was used to analyze and plot PCA graphs, volcano graphs, heatmaps, and KEGG enrichment pathways. SBGNview was utilized to perform Reactome pathway analysis between a pair of comparison groups. The gene ontology (GO) enrichment map was generated by inputting the gene set enrichment analysis (GSEA) result file into the EnrichmentMap (Reimand et al. 2019) App found in Cytoscape software. A p-value cutoff of 0.05 and an FDR Q-value cutoff of 0.1 was selected for stringency and to keep the enrichment map analysis manageable.

### Immunostaining

Eyeballs obtained from the mice were rinsed in PBS and a small incision at the limbus of the cornea was made before fixing them in FAS eye fixation solution for 1 h. The intact cornea was extracted and fixed again for another 1 h. The fixed corneas were placed into a 96-well plate and blocked and permeabilized (0.2% Triton-X and 2% BSA) at room temperature for 1 h. Rabbit anti-Tuj1 antibody (ab18207, Abcam) was added, and the corneas were incubated at 4 °C overnight on a shaker. Following the primary antibody incubation, the corneas were rinsed with PBS thrice and goat anti-rabbit AF488 (A-11008, Invitrogen) secondary antibody was added for incubation at room temperature for 1 h. The corneas were cut into a petal-like shape under a microscope, spread onto a glass slide, and covered with a coverslip after adding a mounting medium.

### Statistical analysis

Statistical analysis and graphical representation of the experimental data were performed using SPSS and GraphPad Prism 9 software respectively. For normally distributed data with homogenous variances, one-way analysis of variance (ANOVA) with the Tukey post hoc test was used for comparisons among multiple groups. For data that were not normally distributed or did not have homogenous variances, the Kruskal–Wallis H test with Mann–Whitney U post hoc test was used for comparisons among multiple groups. All data were presented as mean ± standard deviation (SD). A significance level of p < 0.05 was considered statistically significant, denoted as *p < 0.05, **p < 0.01, ***p < 0.001. ‘N.S.’ indicated no statistical significance.

## Results

### BAK insult led to morphological and pathological changes in the ocular surface of mice

**Fig. 1:**
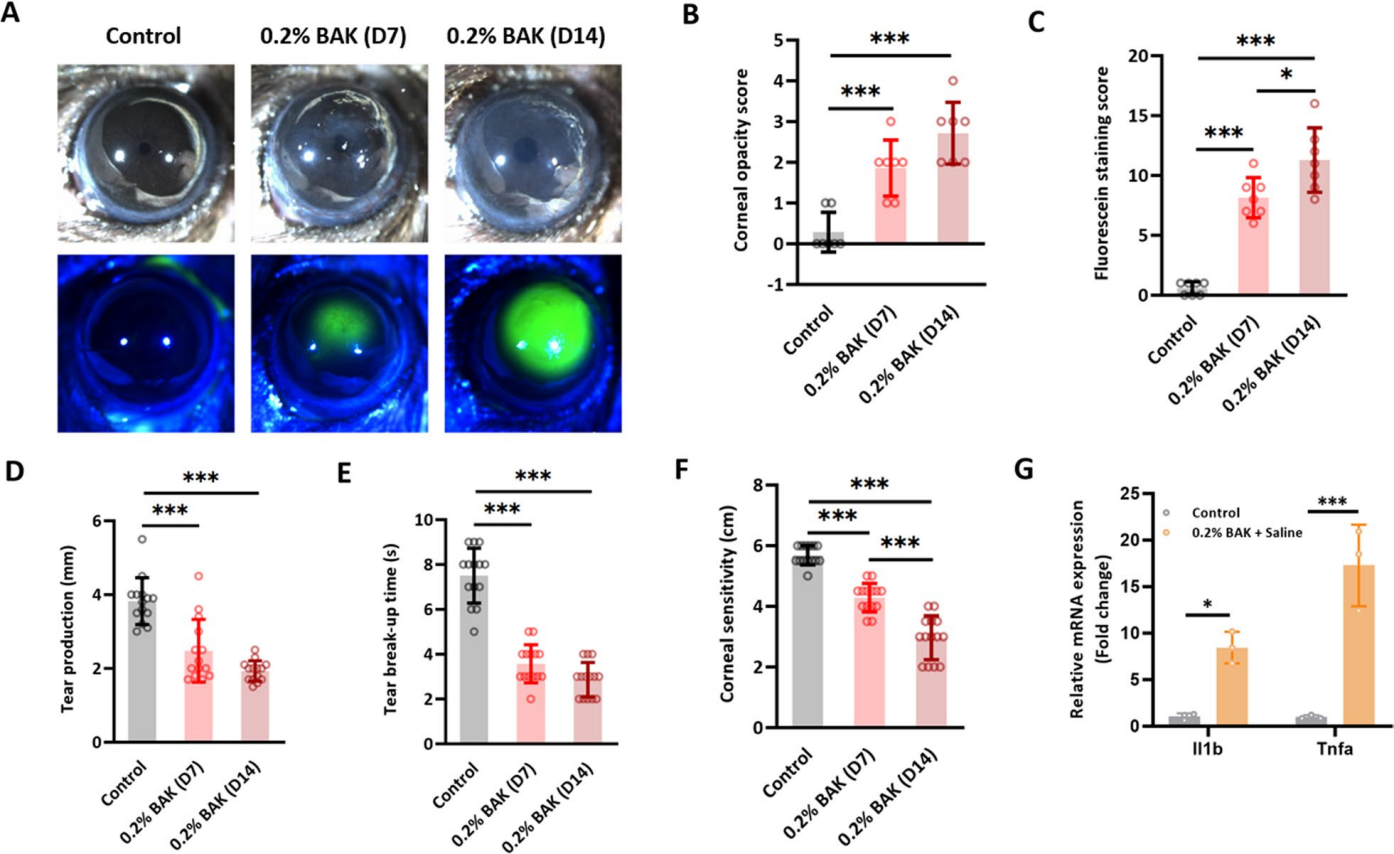
BAK insult led to morphological and pathological changes in the ocular surface of mice. **A** Representative close-up images of the mice cornea showing the change in opacity and fluorescein sodium staining pattern over time following BAK-induced injury. **B** and **C** are the corresponding scoring graphs; n = 7 cornea measurements per group. Besides evaluating morphological changes, the mice’s ocular surface was also subjected to functionality tests which assessed **D** tear production; n = 14 cornea measurements per group, **E** tear break-up time; n = 14 cornea measurements per group and **F** corneal sensitivity; n = 14 cornea measurements per group. **G** Preliminary qPCR screening of the cornea on Day 14 post-BAK injury revealed heightened expression of inflammatory genes such as *Il1b* and *Tnfa*; n = 3 experimental repeats. *p < 0.05, ***p < 0.001. All data were presented as mean ± SD

We first evaluated the effects of BAK on mice corneas. Based on previous studies (Xiao et al. 2012; Zhang et al. 2020), a concentration of 0.2% BAK was adopted. Obvious damage to the morphology and functionality of the ocular surface was observed when BAK was administered consecutively for 14 days. Physical changes to the cornea included increased opacity (Fig. 1A and B) as well as elevated fluorescein sodium staining (Fig. 1A and C) over time, indicating a progressive deterioration in the structural integrity of the cornea. Additionally, there was a significant reduction in tear quantity (Fig. 1D), tear break-up time (Fig. 1E), and corneal sensitivity (Fig. 1F) as the injury progressed. Besides that, BAK is known to induce inflammation in the cornea, with a remarkable upregulation in immune-related genes such as Il1b, Tnfa, Il1, Il12, Il10 and so on (Goldstein et al. 2021; Barabino et al. 2014). Therefore, the gene expression of Il1b and Tnfa was evaluated in this study to validate that BAK was indeed functioning. Specifically, when RNA was extracted from the injured corneas and amplified using qPCR, it was observed that the expression of inflammatory genes Il1b and Tnfa, both known to be induced by BAK (Wu et al. 2023; Launay et al. 2016), were highly upregulated (Fig. 1G), as compared to the control group at Day 14 post administration. Taken together, these results indicated that BAK was functioning and led to adverse outcomes on the ocular surface.

### Administration of BAK resulted in the upregulation of inflammatory responses in the cornea by RNA-seq analysis

**Fig. 2:**
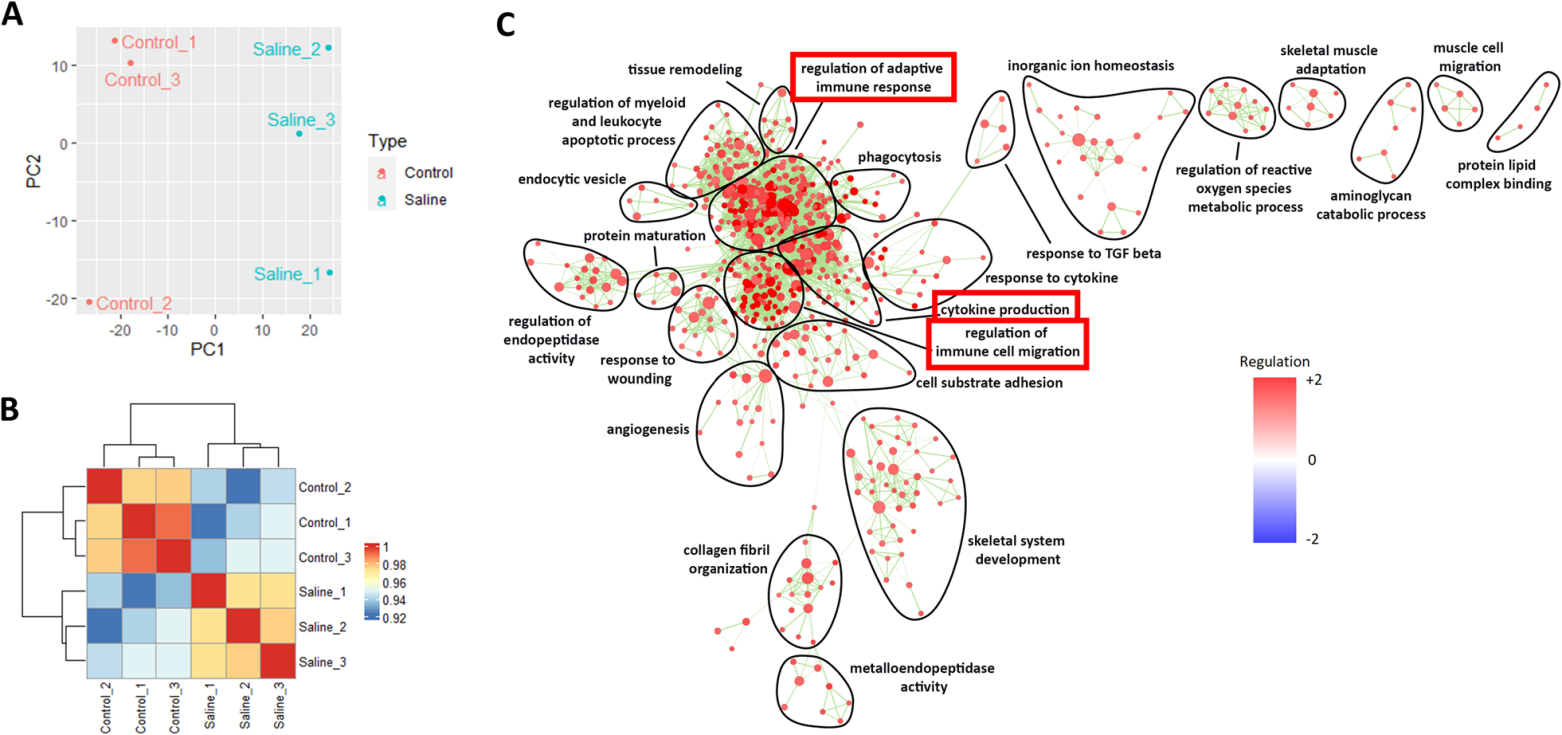
Administration of BAK resulted in the upregulation of prominent inflammatory responses in the cornea by RNA-seq analysis. **A** PCA and **B** correlation plots showed a clear genetic distinction between the corneas in normal animals and untreated BAK-injured animals. **C** Enrichment map generated through GSEA analysis and arranged based on GO pathways. It was observed that the corneal injury involved mainly adaptive immune response, immune cell migration, and cytokine production. All bioinformatics analyzes were performed with n = 3 samples per group

As not much was known about the extent of transcriptional differences exerted by BAK, we next proceeded to perform RNA-seq and compared the differentially expressed genes between corneas treated with only saline and uninjured corneas. Based on PCA and correlation plots (Fig. 2A and B), a clear distinction between these two groups was observed. A corresponding large number of genes were differentially expressed (Additional file 1: Figure S1A and S1B) and KEGG pathway analysis showed that the ‘cytokine-cytokine receptor interaction’ was the most enriched pathway (Additional file 1: Figure S1C). Accordingly, when an enrichment map based on GO classifications was generated, it was observed that huge node clusters, namely ‘regulation of adaptive immune response’, ‘regulation of immune cell migration’, and ‘cytokine production’ were highly upregulated (Fig. 2C). Therefore, these results indicated that administration of 0.2% BAK mainly led to sustained inflammation.

### UCS and UCPRP notably improved the morphology and pathological changes of the ocular surface following BAK insult in mice

**Fig. 3:**
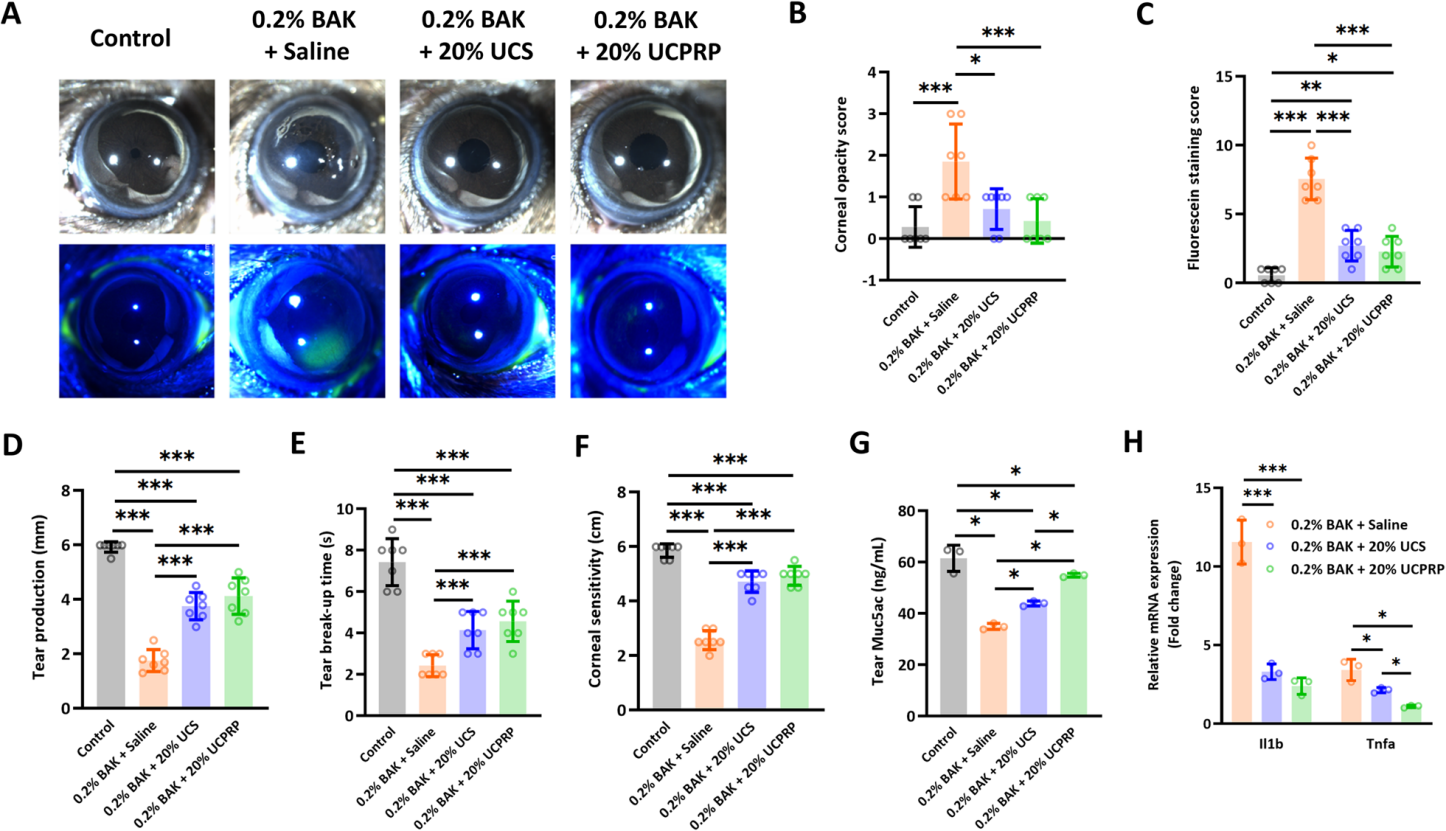
UCS and UCPRP notably improved the morphology and pathological changes of the ocular surface following the BAK insult in mice. **A** Representative close-up images of the BAK-injured mice cornea showing the recovery in opacity and fluorescein sodium staining pattern over time after UCS and UCPRP treatment. **B** and **C** are the respective scoring graphs; n = 7 cornea measurements per group. Additionally, the **D** tear production; n = 7 cornea measurements per group, **E** tear break-up time; n = 7 cornea measurements per group, **F** corneal sensitivity; n = 7 cornea measurements per group, **G** tear *Muc5ac* levels; n = 3 experimental repeats, displayed marked restoration that was close to pre-injury levels. These results also corresponded to a **H** significant downregulation of *Il1b* and *Tnfa at Day 10 post treatment*; n = 3 experimental repeats. *p < 0.05, **p < 0.01, ***p < 0.001. All data were presented as mean ± SD

We next administered 20% UCS and UCPRP and found that these treatments led to marked morphological recovery in mice cornea following BAK-induced injury, which was demonstrated through the reduction of corneal opacity (Fig. 3A and B) and fluorescein sodium staining (Fig. 3A and C). Furthermore, the amount of tear production (Fig. 3D), tear break-up time (Fig. 3E), corneal sensitivity (Fig. 3F) and tear *Muc5ac* levels (Fig. 3G) also showed significant improvements as compared to the saline-treated corneas, though their measurements were not completely restored to pre-injury levels. Additional RNA studies by qPCR assay revealed that these observations corresponded with a decrease of *Il1b* and *Tnfa* in both UCS and UCPRP-treated corneas (Fig. 3H). Together, these results showed that UCS and UCPRP can improve the morphology and pathological changes of the ocular surface following BAK insult.

### Both UCS and UCPRP treatment led to significant downregulation of inflammatory cytokines involved in cytokine-cytokine receptor interaction and Il-17 signaling pathway

**Fig. 4:**
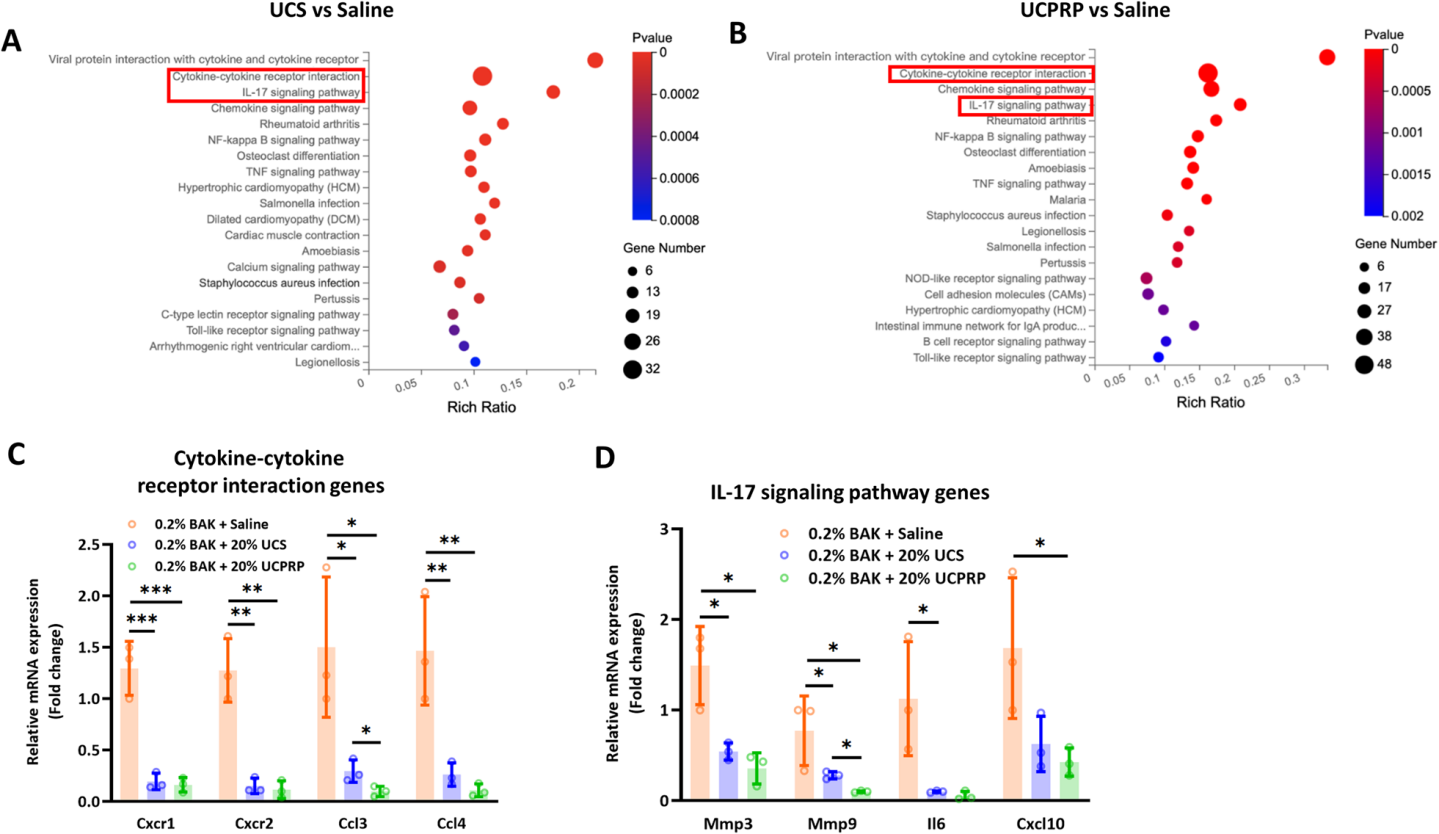
Both UCS and UCPRP treatment led to significant downregulation of inflammatory cytokines involved in cytokine-cytokine receptor interaction and the Il-17 signaling pathway. KEGG enrichment analysis of both **(A)** UCS-treated corneas and **(B)** UCPRP-treated corneas. In both of these analyzes, the cytokine-cytokine receptor interaction and IL-17 signaling pathway were found to be highly regulated. Regulation of some of the genes corresponding to these pathways **(C, D)** was further validated with qPCR. All bioinformatics analyzes were performed with n = 3 samples per group

In order to find out the transcriptional changes induced by UCS and UCPRP, we next performed a similar analysis comparing UCS and UCPRP-treated corneas against saline-only treated corneas. PCA and correlation plots revealed that, unlike the comparison between saline-treated and normal corneas (Fig. 2A and B), the separation between the UCS-treated and saline-treated groups was relatively less distinct (Additional file 1: Figure S2A and S2B). This was depicted in the volcano plot and heatmap where besides containing lesser differentially expressed genes, most were also downregulated (Additional file 1: Figure S2C and S2D). Additionally, the enrichment map based on GO classifications showed that UCS treatment resulted in a plethora of pathway downregulation such as ‘leukocyte migration’, ‘ossification’, and ‘response to wounding’ (Additional file 1: Figure S2E). Further analysis of KEGG pathway changes showed that UCS treatment led to a notable downregulation of the ‘cytokine-cytokine receptor interaction’ pathway as well as the ‘IL-17 signaling pathway’ (Fig. 4A). In UCPRP-treated corneas, the separation between the UCPRP-treated and saline-treated group in the PCA and correlation plots were also not distinct (Additional file 1: Figure S3A and S3B) and the differentially expressed genes were mostly downregulated (Additional file 1: Figure S3C and S3D) as well. Besides that, the enrichment map based on GO classification revealed downregulation of immune-related pathways such as ‘regulation of chemotaxis and immune cell migration’, ‘innate immune response and interferon production’, and ‘antigen processing and presentation’ (Additional file 1: Figure S3E). Similar to UCS-treated samples, KEGG analysis in UCPRP-treated samples also showed marked regulation of the ‘cytokine-cytokine receptor interaction’ pathway and ‘IL-17 signaling pathway’ (Fig. 4B). Given that these two pathways were highly downregulated following UCS and UCPRP treatment, we proceeded to validate the expression of some of the genes, namely *Cxcr1, Cxcr2, Ccl3, Ccl4, Mmp3, Mmp9, Il6, and Cxcl10*, present in these pathways (Additional file 1: Figure S2F, S2G, S3F, and S3G) using qPCR and found that all of them were indeed significantly downregulated (Fig. 4C and D). Thus, these results indicated that UCS and UCPRP reduced inflammation through downregulating cytokine receptor interactions and Il-17 signaling in BAK-injured mice cornea.

### UCS and UCPRP treatments led to robust axonal regeneration in the cornea

**Fig. 5:**
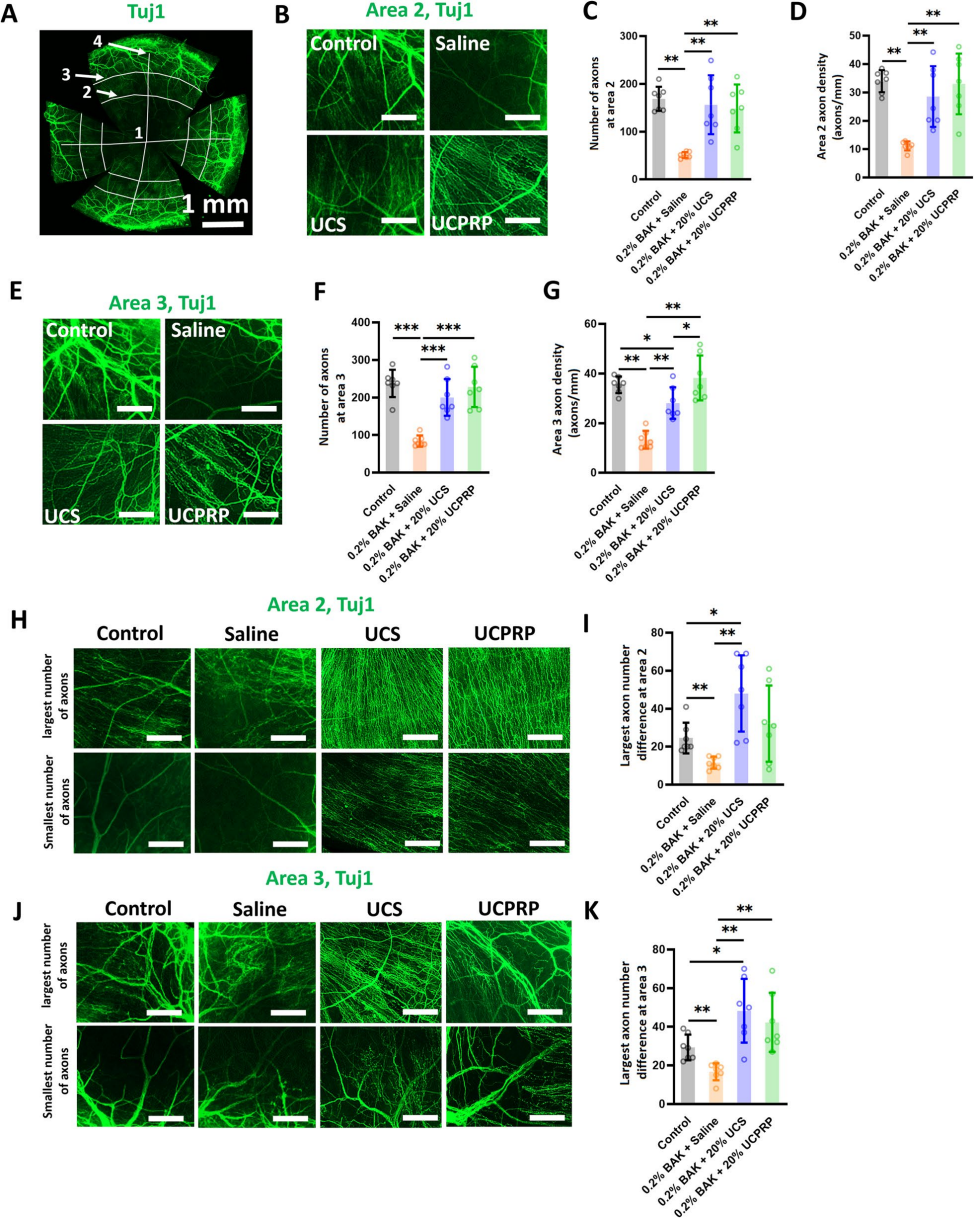
UCS and UCPRP treatments led to robust axonal regeneration in the cornea. **A** Illustration of regional division of a mouse cornea with Tuj1 staining. Area 1 is the middle of the cornea while Area 4 is the limbus. **B** Representative high magnification images of axons in Area 2 with the corresponding **C** total quantified axonal number and **D** axonal density. **E** Representative high magnification images of axons in Area 3 with the corresponding **F** total quantified axonal number and **G** axonal density. **H** Representative high magnification images of axons in Area 2 with the corresponding **I** quantified largest axon difference. **J** Representative high magnification images of axons in Area 3 with the corresponding **K** quantified largest axon difference. *p < 0.05, **p < 0.01, ***p < 0.001. All data were presented as mean ± SD, scare bar = 10 µm for Figure **B**, **E**, **H** and **J**

As we observed significant sensitivity recovery in the cornea, we next proceeded to evaluate the condition of the axons in all experimental groups using immunostaining. According to literature, axons arranged in a whorl-like pattern in the inferocentral cornea as well as their connecting nerve fibres are defined as the sub-basal nerve plexus (Cruzat et al. 2017; Badian et al. 2021). On a normal cornea, axons from the sub-basal nerve plexus were observed to extend from Area 1 to Area 3 (Fig. 5A). However, following BAK injury, we noticed that the number of axons varied widely in Area 1, leaving an unequal baseline level of the remaining axons. Hence, quantification of axons at Area 1 after treatment can be quite variable and inaccurate. On the other hand, the axons in Area 4, where the limbus is located, were observed to be neither damaged nor regenerated. Therefore, only axons in Area 2 and 3 were quantified for rigorous comparison. Results indicated that the total number (Fig. 5B and C) and density (Fig. 5B and D) of axons in Area 2 and 3 (Fig. 5E–G) in both UCS and UCPRP-treated corneas were almost similar to those found in the uninjured corneas. Besides that, when we compared the number of axons between the four regions, it was observed that the regenerated axons in UCS-treated samples were notably skewed towards certain region of the cornea and not uniformly distributed. Specifically, in the upper row of Fig. 5H, denoted as the largest number of axons, a more robust nerve infiltration was observed whereas in the lower row, the number of nerve fibres infiltrated to Area 2 was much less, indicating a skew in a certain quadrant versus the other at the same Area. Similar phenomena were observed in Area 3 as well (Fig. 5J). Although some UCPRP-treated corneas showed a similar trend, there were overall not statistically different from the normal cornea (Fig. 5H–K). Together, these findings confirmed that robust axonal regeneration likely played a huge role in the functional recovery of the cornea.

### Axonal enhancers were present throughout injury and treatment

**Fig. 6:**
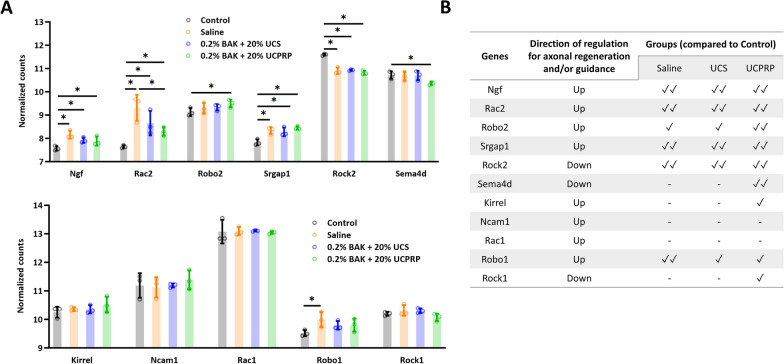
Axonal enhancers were present throughout injury and treatment. **A** Normalized counts of axon enhancers and **B** the corresponding qualitative assessment of each group’s potential in inducing axonal regeneration

To further investigate how UCS and UCPRP treatment led to axonal regeneration, we utilized the reactome pathway database in SBGNview and screened for axonal pathways that were regulated during treatment. In total, 4 pathways, namely ‘Axonal guidance by semaphorins’, ‘Axon guidance by Slit_Robo’, ‘Axonal growth stimulation’ and ‘Sema4D induced cell migration and growth-cone collapse’ (Additional file 1: Figure S4) were found to be relevant for the analysis. The genes present in these pathways were then listed in a table and a qualitative assessment of each of the group’s potential in inducing axonal regeneration based on the gene’s normalized counts were conducted (Fig. 6A and B). Of note, the assessment revealed that some of these axonal enhancers were already present during BAK injury, and their regulation was nearly identical to those induced by UCS and UCPRP treatment. Given that the major differences between the injury (BAK) and treatment (UCS and UCPRP) groups lay in their inflammatory responses, these findings suggested that excessive inflammation inhibited axonal regeneration.

### Cord blood-derived biologics did not reduce inflammatory responses in BAK-injured corneas to pre-injury levels

**Fig. 7:**
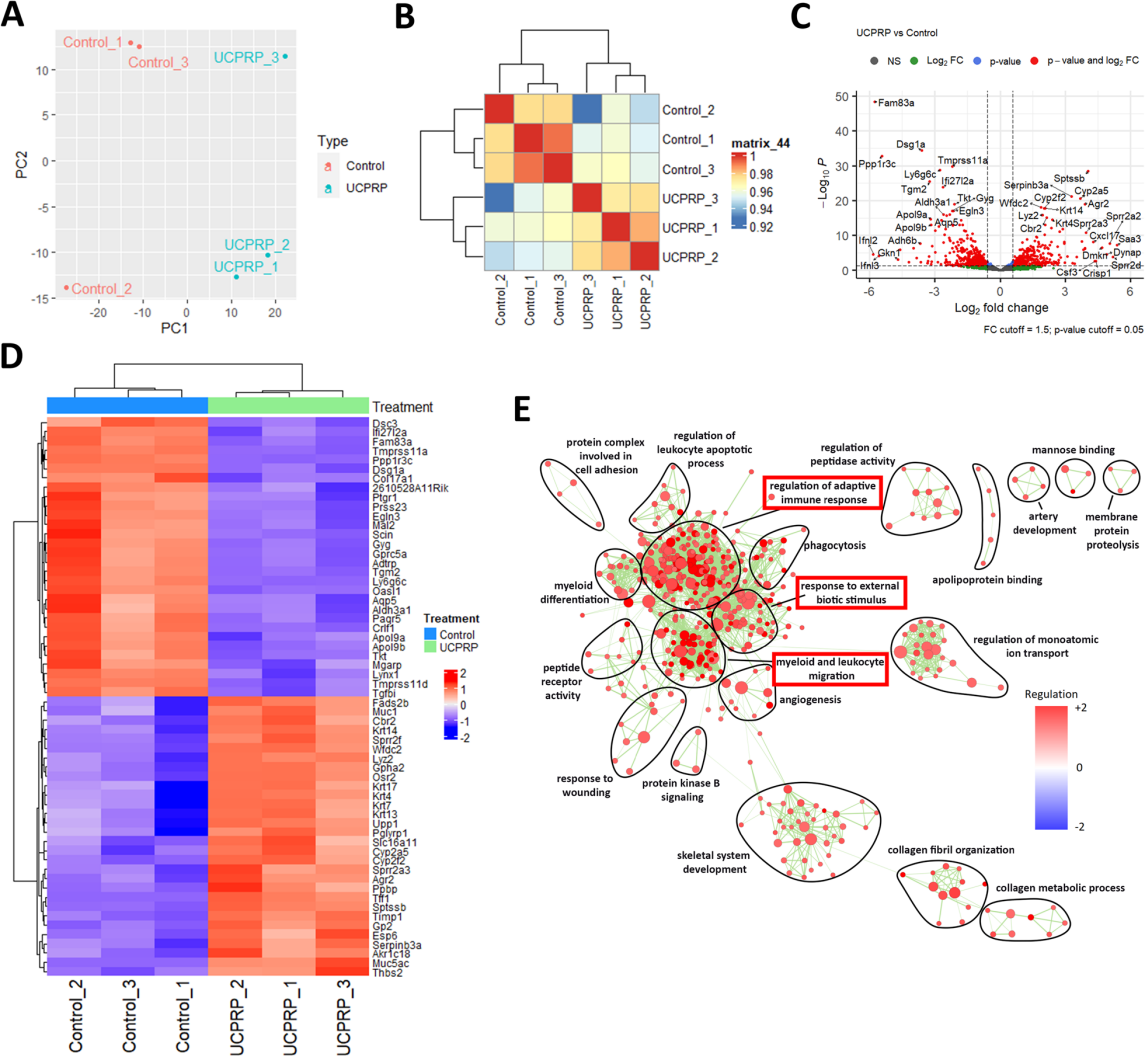
Detailed analysis of the clinical effectiveness of UCPRP treatment in BAK-induced corneal injury. **A** PCA and **B** correlation plots show the comparison between UCPRP-treated and normal corneas. **C** Volcano plot and **D** heat map of the top regulated genes following UCPRP treatment. **E** GO pathway analysis of UCPRP-treated corneas vs control corneas show that many pathways remain upregulated. All bioinformatics analyzes were performed with n = 3 samples per group

Although it was found that both UCS and UCPRP can reduce similar inflammatory responses, it is not known how these regulations compare to the normal cornea. Therefore, we next analyzed the gene and pathway changes between UCPRP-treated corneas and normal corneas. Accordingly, a clear distinction between sample groups can be observed from PCA and correlation plots (Fig. 7A and B). This was corroborated by the large amounts of differentially expressed genes in both the volcano plot and heatmap (Fig. 7C and D). When we generated the GO enrichment map, it appeared that many of the immune response clusters such as regulation of adaptive immune response and immune cells migration remained upregulated, indicating that while UCS and UCPRP treatment could lead to their downregulation, their levels remained relatively high compared to a normal cornea (Fig. 7E). In sum, these results showed that even though most of the dysregulated pathways were not restored to pre-injury levels, UCS and UCPRP were still able to promote axonal regeneration.

### Neuropeptide Y was involved in the modulation of inflammation

**Fig. 8:**
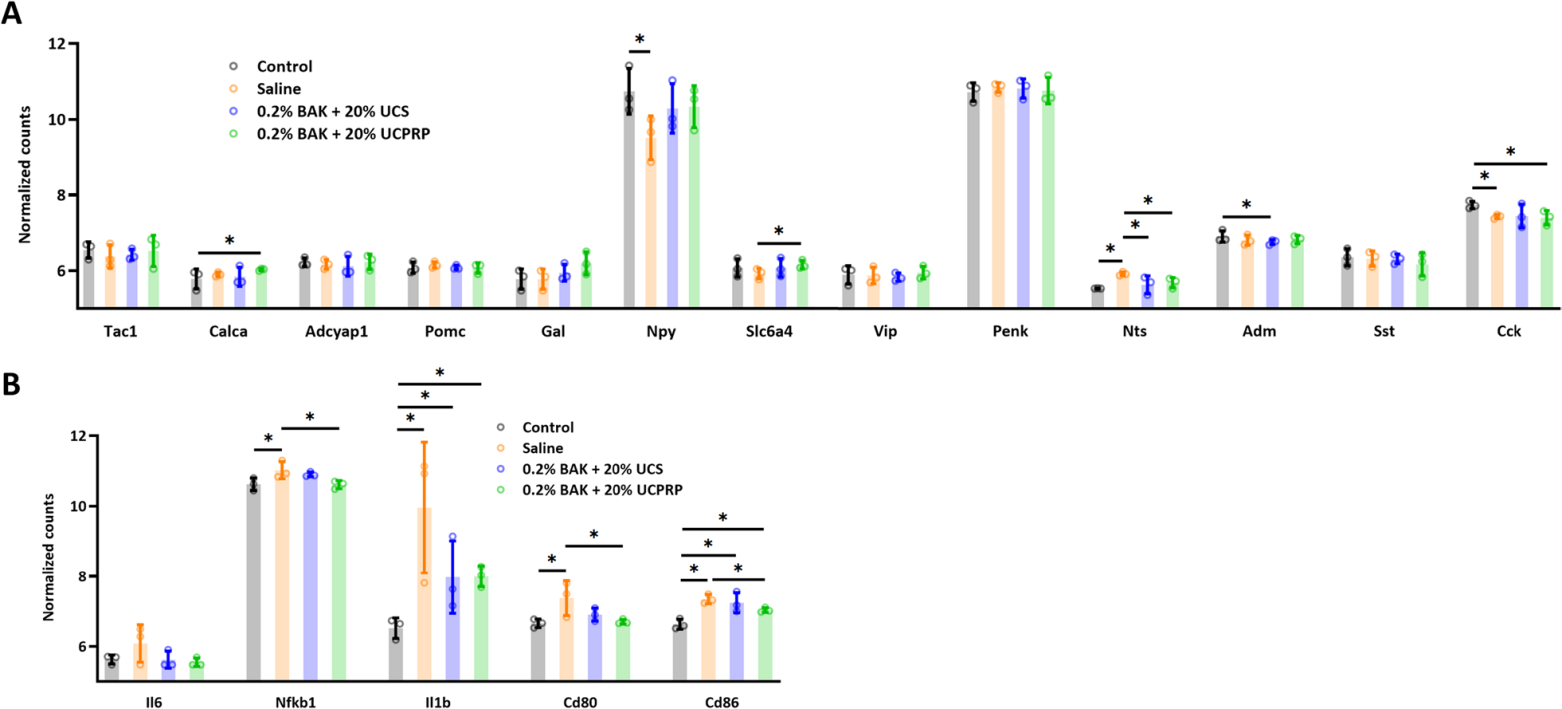
Neuropeptide Y was involved in the modulation of inflammation. **A** Normalized counts of known neuropeptides in the cornea axons. **B** Normalized counts of the downstream targets of Npy

As cornea axons are modulated by a variety of neuropeptides, we proceeded to plot their normalized counts for comparison between all groups (Fig. 8A). It was found that Neuropeptide Y (*Npy*), upon evaluating its downstream targets (Fig. 8B), was involved in the modulation of inflammatory pathways following UCS and UCPRP treatment as well.

## Discussion

Umbilical cord blood derivatives such as serum and platelet-rich plasma are potent therapeutics due to the heightened amount and quality of growth factors present as compared to autologous serum and platelet-rich plasma (Samarkanova et al. 2020). However, in-depth characterization of their treatment effects is not well documented and mostly conjectures based on the effects of individual growth factors detected. This study has two major impacts. First, we validated the feasibility of using UCS and UCPRP in treating severe BAK-injured mice cornea. Second, we showed that both UCS and UCPRP were able to promote robust axonal regeneration in the BAK-injured mice cornea.

The harmful effects of BAK on the ocular surface were well documented (Vitoux et al. 2020; Kahook and Noecker 2008; Kim et al. 2016; Goldstein et al. 2022). Combining the results from animal studies and RNA-seq analysis, we were able to obtain a multifaceted perspective on how BAK could be detrimental to the ocular surface. Specifically, we observed a significant elevation of fluorescein sodium staining and opacity in the cornea as well as a marked reduction of tear production, tear break-up time, tear *Muc5ac* levels, and corneal sensitivity (Fig. 1). These observations corresponded with the upregulation of inflammatory genes involved in the ‘regulation of adaptive immune responses’, ‘regulation of immune cell migration’, ‘cytokine production’ and ‘cytokine-cytokine receptor interaction’ pathways. These results were consistent and more comprehensive than previous studies where they observed elevated levels of lymphocyte infiltration (Kahook and Noecker 2008; Liang et al. 2012), inflammatory markers such as *Ccl2, Il6, Mif,* and *Tnfa* (Vitoux et al. 2020; Kim et al. 2015) as well as cellular apoptosis. (Kim et al. 2016; Pauly et al. 2011)

Following the administration of UCS and UCPRP, we observed widespread gene downregulation. Specifically, using qPCR, we demonstrated reduced Tnfa mRNA expression at 10 days post-UCS treatment (Fig. 3H), aligning with a prior research indicating a notable decrease in Tnfa mRNA level in mice with corneal alkali burns at 7 days post UCS treatment compared to PBS (Han et al. 2019). Besides that, Through KEGG analysis, both UCS and UCPRP treatment was found to lead to the downregulation of the ‘cytokine-cytokine receptor interaction’ pathway and the ‘IL-17 signaling pathway’ (Fig. 4). We also validated some of the genes found in these pathways, specifically *Cxcr1, Cxcr2, Ccl3, Ccl4, Mmp3, Mmp9, Il6,* and *Cxcl10*. *Cxcr1* and *Cxcr2* are chemokine receptors that are expressed in neutrophils and monocytes (Molczyk and Singh 2023). Accordingly, the downregulation of both of these chemokine receptors suggested that both of these cell types were reduced in the UCS and UCPRP-treated samples. *Ccl3* is a chemotactic chemokine secreted by macrophages that can recruit other macrophages and lymphocytes via the *Ccr1* or *Ccr5* receptor (Bhavsar et al. 2015; Gladue et al. 2010). *Ccl4* is also a chemoattractant but can only act via the *Ccr5* receptor, which is expressed by T cells, immature dendritic cells (DCs), monocytes, and natural killer (NK) cells (Ha et al. 2017). Therefore, their downregulation suggested reduced levels of chemokines for attracting lymphocytes. Macrophages and neutrophils are both sources of *Mmp3* and *Mmp9* (Gibbs et al. 1999a, 1999b*).* However, fibroblasts and epithelial cells can produce them as well when exposed to pro-inflammatory factors (Warner et al. 2004). Both of these matrix metalloproteinases (*MMPs*) have been shown to facilitate inflammation as their functional deletion and downregulation led to failed and alleviated inflammatory responses respectively (Warner et al. 2004; Wang et al. 1999). Based on our results, although it is not known if the downregulation of *Mmp3* and *Mmp9* is a result or cause of reduced inflammation, the outcome remained beneficial for corneal healing. Besides that, *Il6* is a well-known pleiotropic cytokine secreted by a large variety of cells that participates in inflammation and tissue homeostasis while *Cxcl10* is also a chemokine that binds to *Cxcr3* to activate and recruit leukocytes such as T cells, monocytes, and NK cells (Amo et al. 2022; Vazirinejad et al. 2014). To our knowledge, there were no studies that documented the downregulation of both of these pathways following UCS and UCPRP treatment. However, we found an earlier study that predicted the regulation of the IL-17 signaling pathway using platelet-rich plasma (PRP), suggesting that our results were in agreement with the literature (Amo et al. 2022). Taken together, these analyzes suggested that the amelioration of inflammatory responses by UCS and UCPRP could largely be attributed to the reduction of innate immune cells, the decrease in chemoattractants, which likely reduced the activation and recruitment of adaptive immune cells, as well as the lessening of pro-inflammatory *MMPs*.

Besides that, the restoration of corneal sensitivity was largely due to the robust axonal recovery observed in UCS and UCPRP treatment groups. Although it was shown that the regulation of key axonal guidance and/or regeneration genes (*Ngf, Rac2, Robo2, Srgap1, Rock2, Sema4d*) (Mandemakers and Barres 2005; Hivert et al. 2002; Lindsay 1988; Koch et al. 2014; Harris et al. 2020) played a role in their recovery, the reduction of extreme extents of inflammatory responses was most likely the main contributor in enabling it. In this study, the direction of regulation for the axonal genes in the saline group was mostly comparable to the UCPRP treatment group and exactly similar to the UCS treatment group (Fig. 6B). However, axon regeneration was only observed in UCS and UCPRP groups where inflammation was also found to be dampened, therefore suggesting that certain inflammatory responses might also play a role in axonal recovery. Of note, although the inflammatory response was alleviated in UCS and UCPRP-treated samples, they were still relatively upregulated when compared to normal samples (Fig. 7). This observation was consistent with previous studies, which demonstrated the role of inflammation in promoting axonal regeneration (Mietto et al. 2015; Bollaerts et al. 2017; Leon et al. 2000; Hauk et al. 2010). Additional evidence of how inflammation might be related to axon regeneration was revealed in the prominent downregulation of *Npy*, a neuropeptide expressed by corneal sympathetic nerves (Puri et al. 2022), in the saline group, which corresponded to heightened gene expression of its known downstream targets (*Il6*, *Nfkb1, Il1b, Cd80*, and *Cd86*) (Chen et al. 2020; Ferreira et al. 2011; Ding et al. 2021). This trend was reversed when UCS and UCPRP were administered, indicating that these treatments were able to elevate the expression of *Npy* following BAK injury and subsequently reduce the expression of the immune-related genes. Such interactions where neuropeptides were observed to facilitate crosstalk between the immune and nervous systems on the ocular surface were documented in recent studies (Wu et al. 2022; Lee et al. 2023). However, our findings primarily demonstrated the correlation between inflammatory levels and axonal regeneration in the cornea. While they may potentially describe the neuroimmune axis after UCS and UCPRP treatment, more work elucidating the temporal order of regulation between inflammation and regeneration using techniques with higher resolution such as scRNA-seq or CITE-seq should be conducted.

Lastly, there are some limitations to using UCS, UCPRP, or any other blood-derived biologics for corneal treatment. Firstly, vascular endothelial growth factor (VEGF) is one of the known factors in UCS and UCPRP and a main driver of angiogenesis and neovascularization (Yoon et al. 2007; Murphy et al. 2012). Using UCS or UCPRP for cornea treatment would therefore be ill-advised as it may lead to corneal neovascularization, a condition where blood vessels form on the cornea and cause vision loss. However, as the cornea can actively maintain avascularity, this concern may be more applicable to susceptible patients (Zazzo et al. 2021). Secondly, both UCS and UCPRP are allogeneic and may lead to adverse reactions. Thirdly, blood-derived therapeutics are heterogeneous and may vary during each treatment session. However, based on our current study, we have shown that on average, treatment with UCS and UCPRP resulted in beneficial outcomes. Future clinical studies are required to ensure that the proposed method in this work is, without a doubt, safe for the patients. Furthermore, UCS and UCPRP can also be used in areas where axonal regeneration is required. For example, they can potentially be administered into the vitreous humor for treating some retinal lesions, where the retinal ganglion cells (RGCs) and the nerve fiber axon reside. Last but not least, while we were able to observe beneficial effects of UCS and UCPRP on BAK-injured corneas, our results remain gender-specific. For completeness, future tests will also be conducted on male mice and compared with our current data.

## Conclusion

Through animal studies and RNA-seq analysis, we confirmed that severe BAK-induced injury triggers the upregulation of both innate and adaptive immune responses, resulting in observable morphological and pathological alterations in the cornea. Following the administration of UCS and UCPRP, significant corneal recovery was achieved alongside the suppression of extensive immune pathway activation. Subsequent verification of key genes within two prominent KEGG pathways indicated that this reduction in inflammation likely stemmed from a decrease in immune cell presence, diminished chemoattractant levels, and a decrease in pro-inflammatory MMPs. Additionally, the regulation of pivotal axonal genes and Npy facilitated robust axon recovery, closely correlating with the near-complete restoration of corneal sensitivity. These findings not only underscore the potential of cord blood-derived therapeutics in addressing sustained BAK-induced corneal injuries but also emphasize the critical role of achieving an optimal level of inflammatory response to stimulate axon regeneration.

### Supplementary Information

**Additional file 1. **Supplementary infomation supporting the findings of this work.

## Data Availability

The RNA-seq data that support the findings of this study have been uploaded to the Gene Expression Omnibus (GEO). This section will be updated accordingly once the corresponding accession number has been assigned.
