# Supplementary material for: Cord blood-derived biologics lead to robust axonal regeneration in benzalkonium chloride-injured mouse corneas by modulating the Il-17 pathway and neuropeptide Y
Source: Mol Med. 2024 Jan 3;30:2. doi: 10.1186/s10020-023-00772-w (PMC10763178; doi:10.1186/s10020-023-00772-w)
Supplement: Supplementary file 1 — Additional file 1. Supplementary infomation supporting the findings of this work. [file 10020_2023_772_MOESM1_ESM.docx]

**Table S1. Animal grouping information**


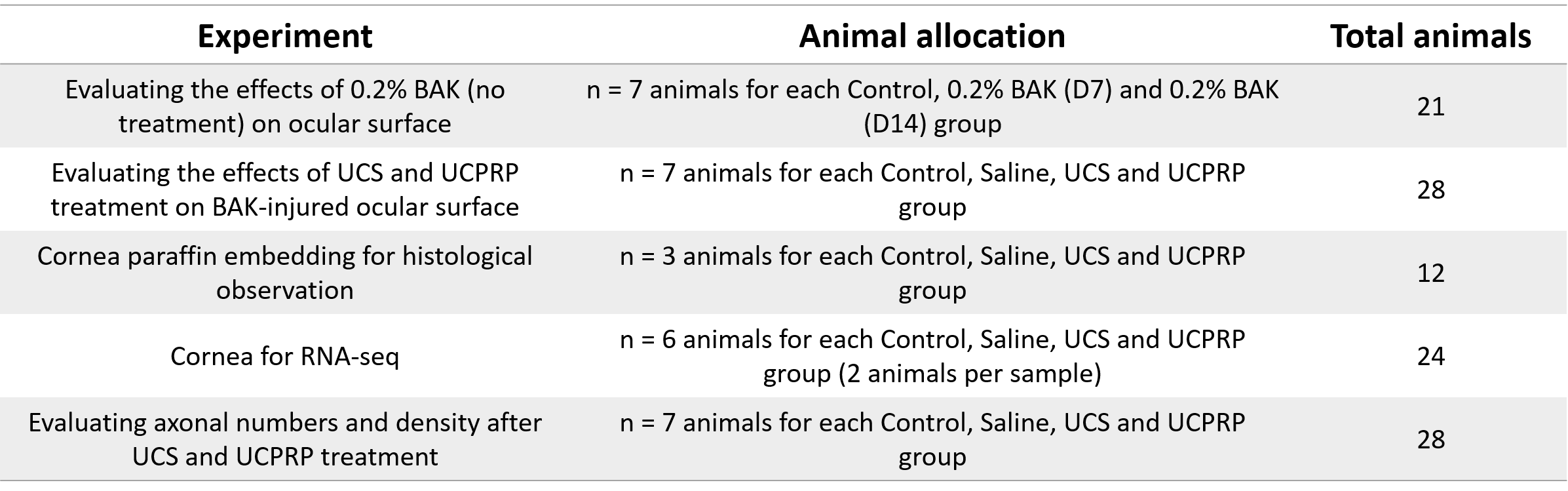


**Table S2. Primer information**


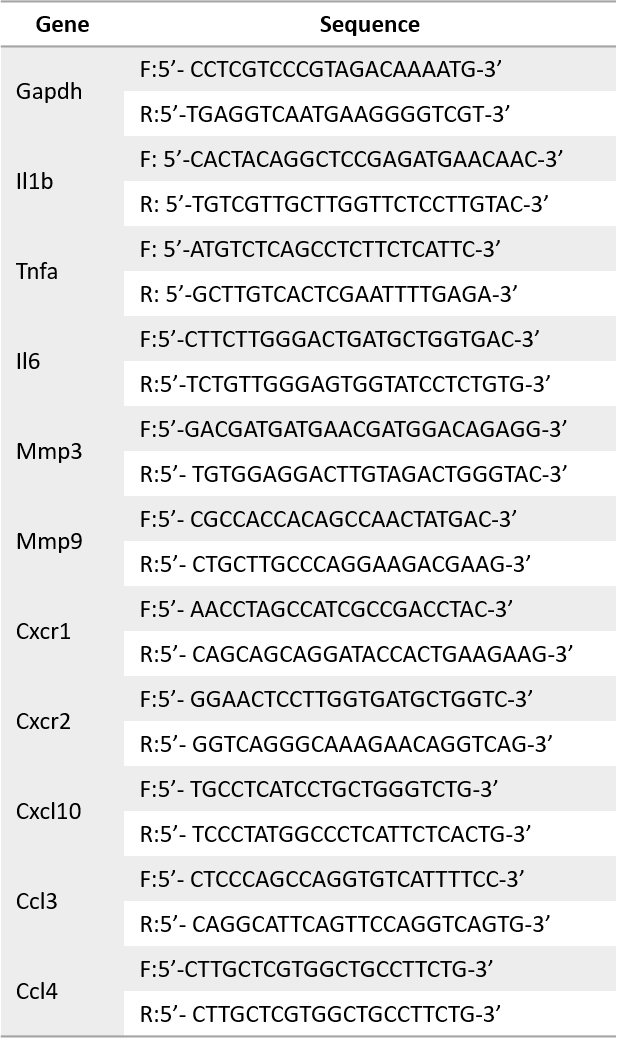


**Table S3. Information and quality of RNA samples utilized in RNA-seq**


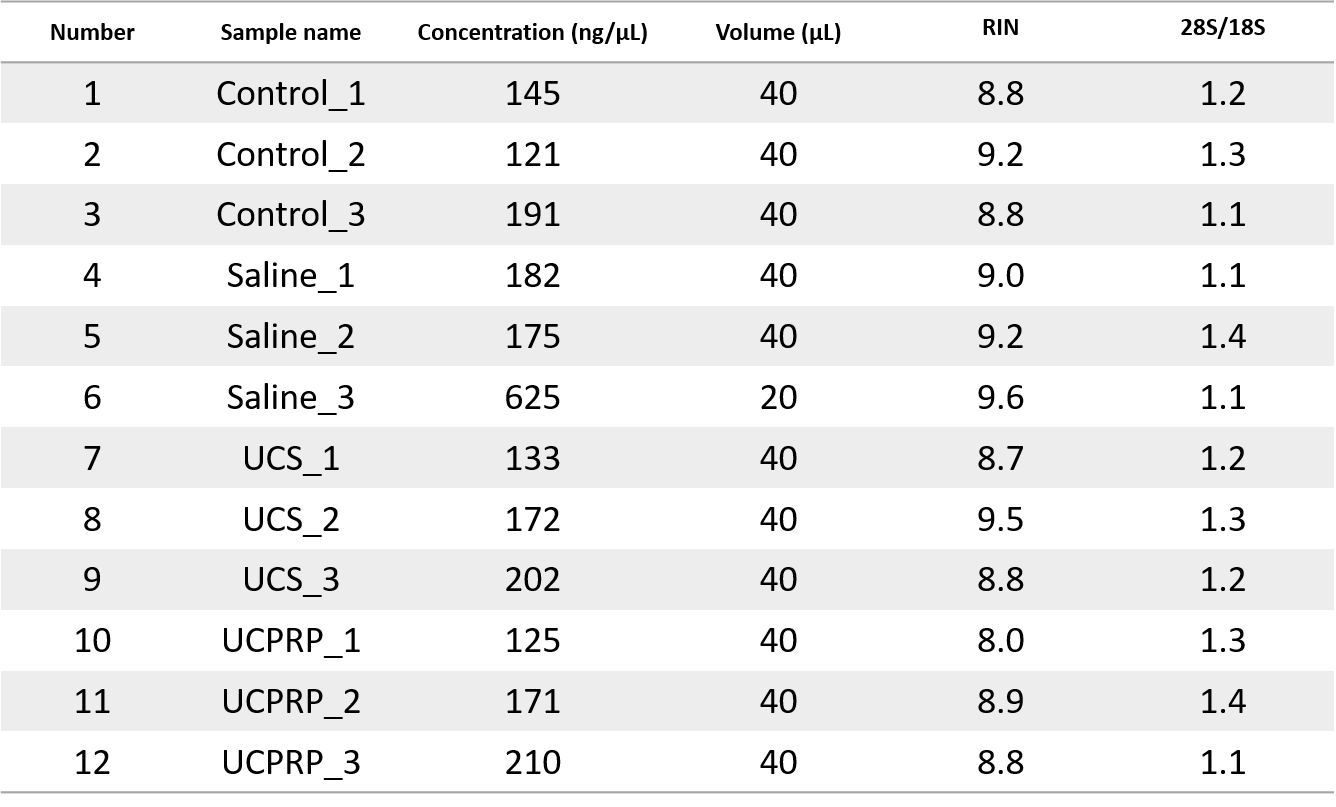


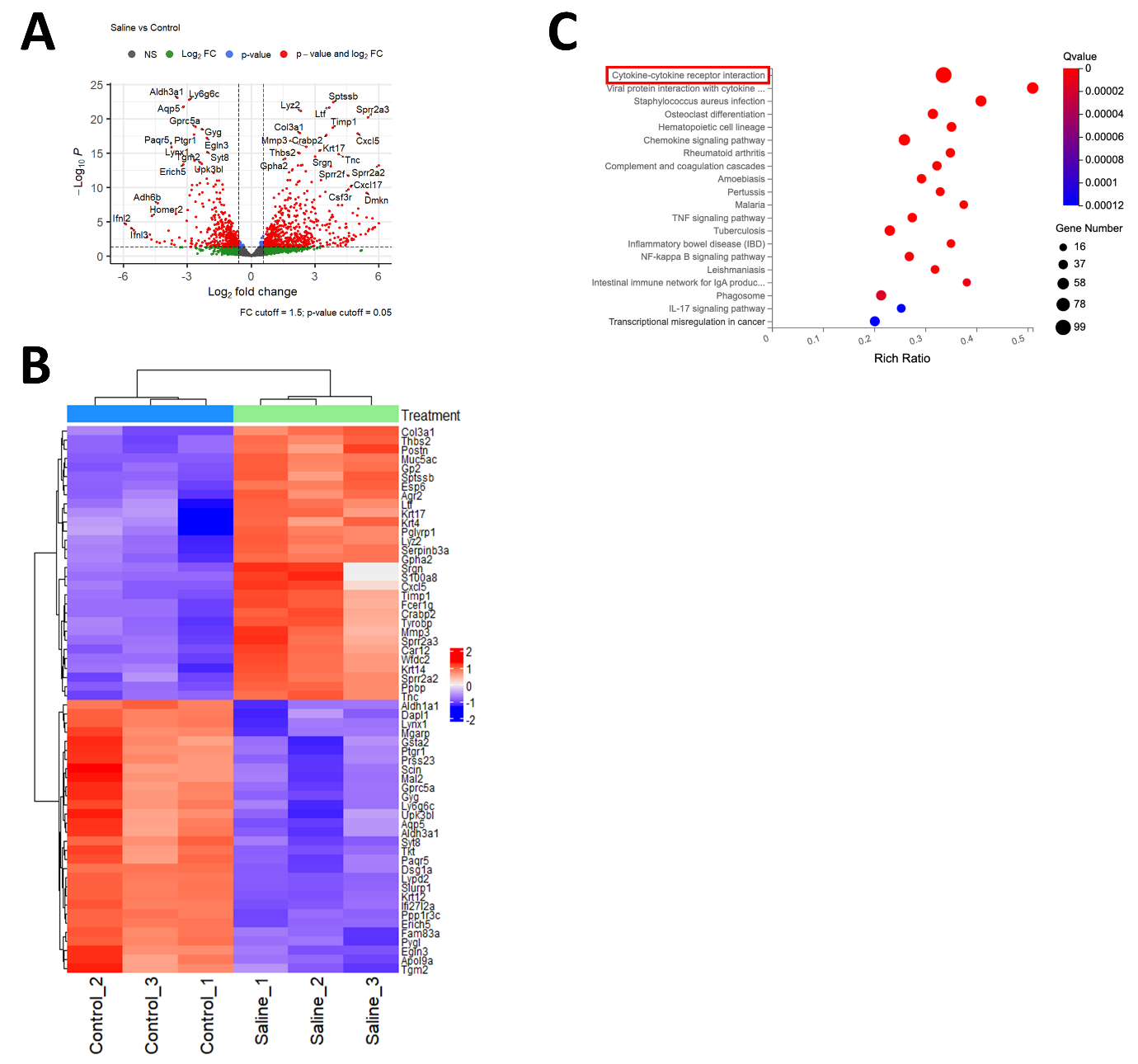


**Figure S1. Notable gene changes and KEGG pathway following BAK insult.** **(A)** Volcano plot of untreated 0.2% BAK-injured mice cornea vs normal mice cornea. **(B)** Heatmap of top regulated genes. **(C)** KEGG enrichment plot depicting the highly enriched pathways 14 days post BAK injury.


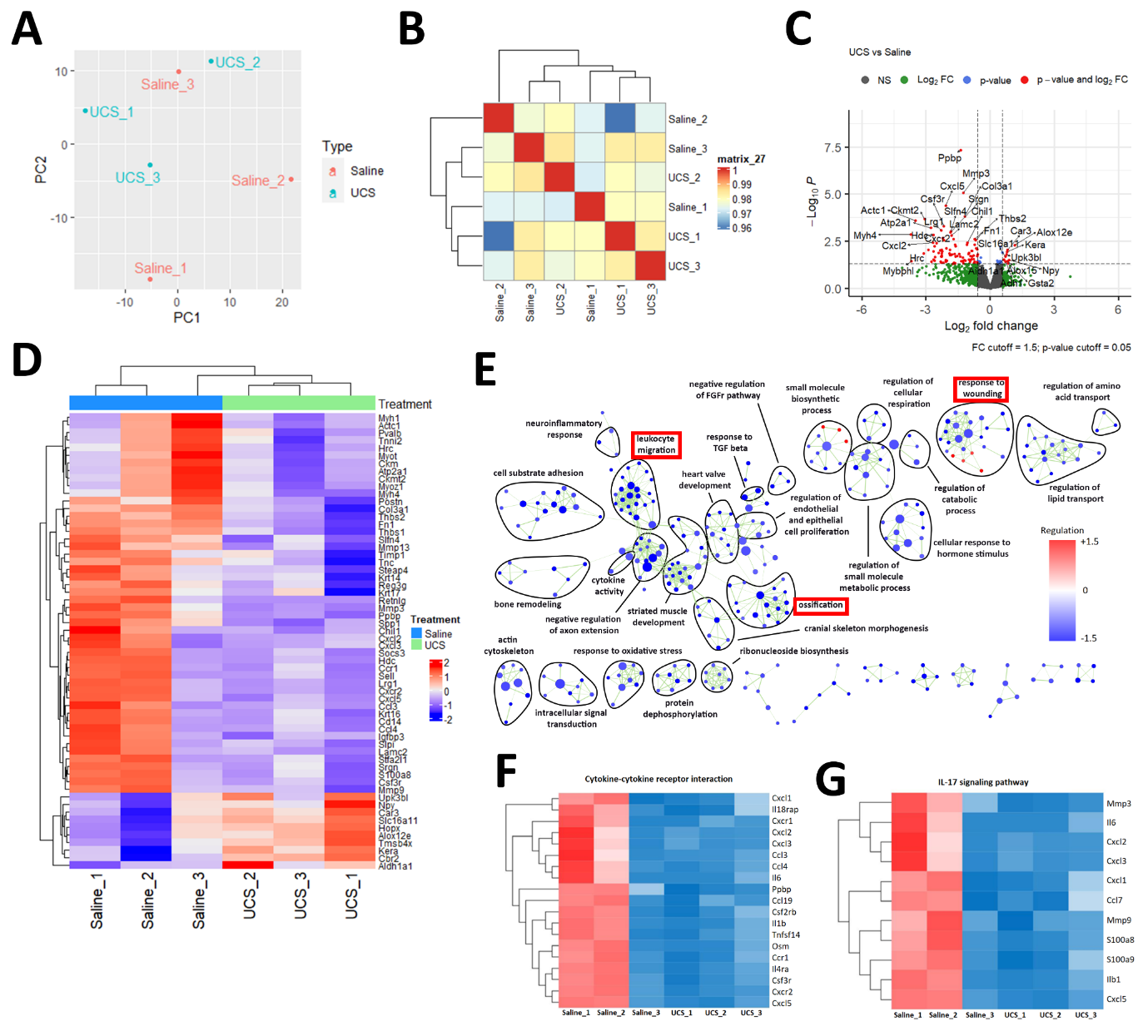


**Figure S2. Detailed analysis of UCS treatment for BAK-induced corneal injury.** **(A)** PCA and **(B)** correlation plots show the comparison between UCS-treated and untreated BAK-injured corneas. **(C)** Volcano plot and **(D)** heat map of the top regulated genes following UCS treatment. **(E)** GO pathway analysis revealed many downregulated pathways. Regulation of the genes within the cytokine-cytokine receptor interaction and IL-17 signaling pathways were presented in heatmaps **(F)** and **(G)** respectively.


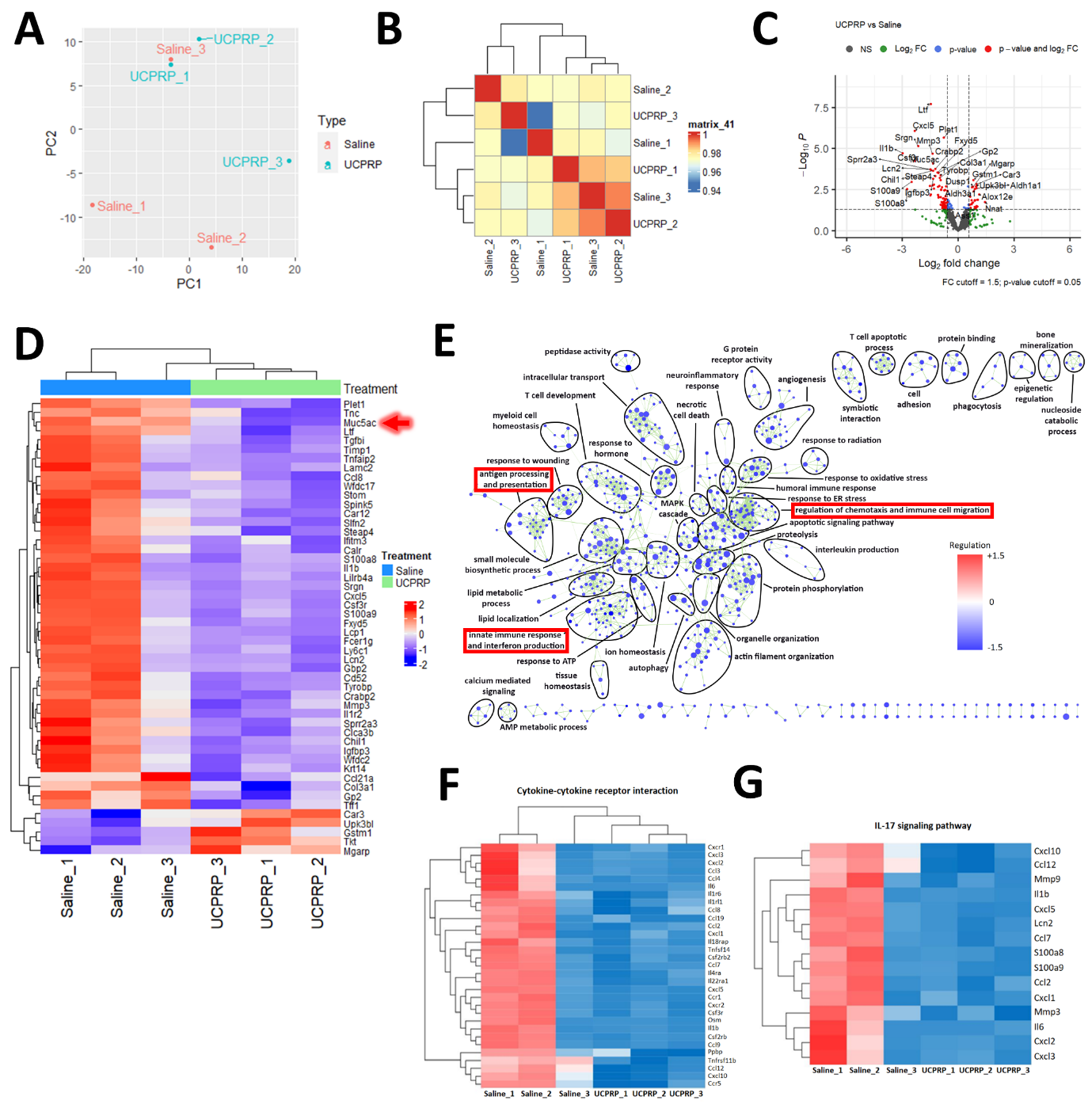


**Figure S3. Detailed analysis of UCPRP treatment for BAK-induced corneal injury.** **(A)** PCA and **(B)** correlation plots show the comparison between UCPRP-treated and untreated BAK-injured corneas. **(C)** Volcano plot and **(D)** heat map of the top regulated genes following UCS treatment. **(E)** GO pathway analysis revealed many downregulated pathways, similar to UCS treatment. Regulation of the genes within the cytokine-cytokine receptor interaction and IL-17 signaling pathways were presented in heatmaps **(F)** and **(G)** respectively.


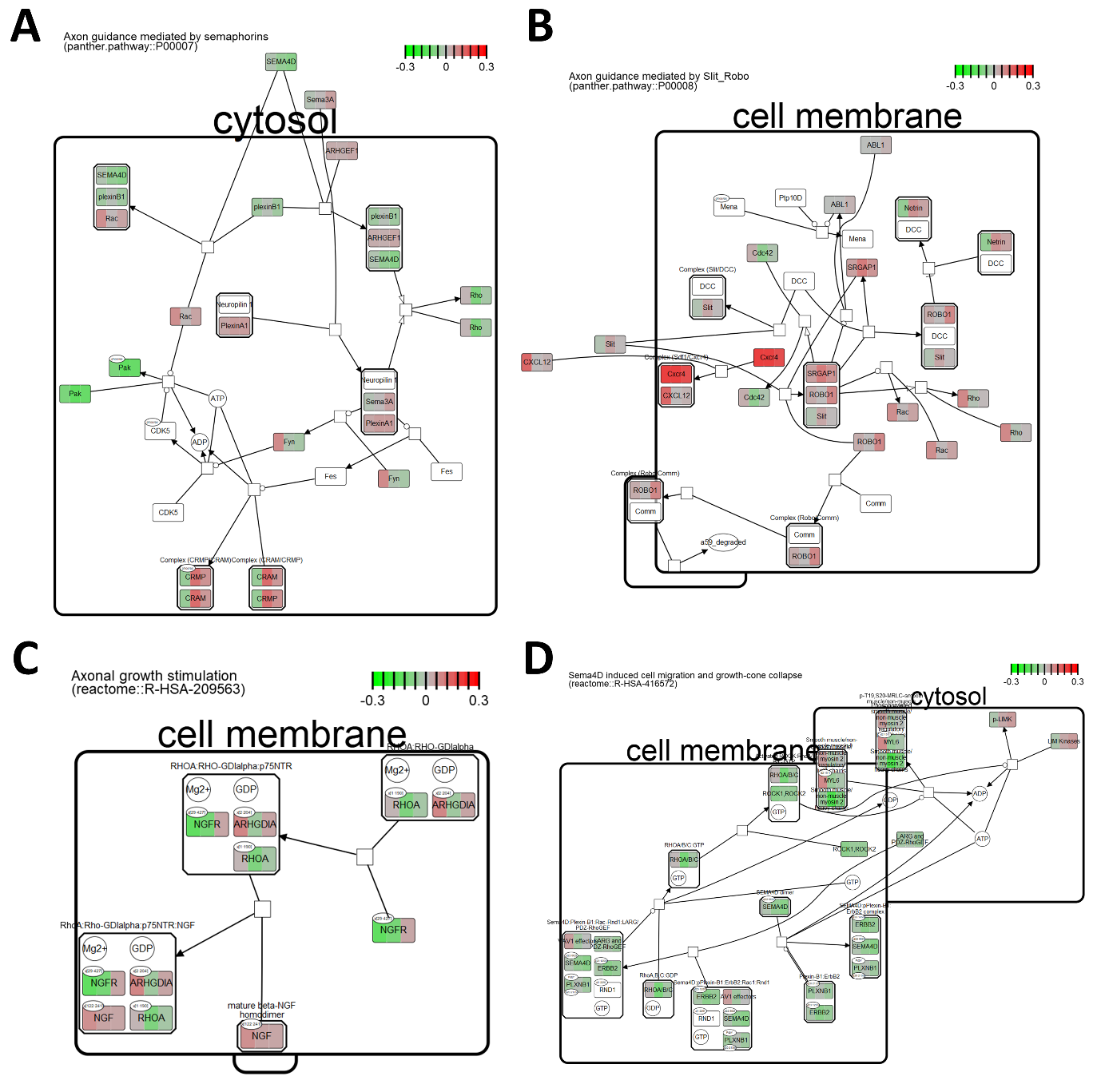


**Figure S4. Axon-related Reactome pathways.** Pathways that were referenced for selecting the relevant genes included **(A)** axon guidance mediated by semaphorins, **(B)** axon guidance mediated by Slit_Robo, **(C)** axonal growth stimulation and **(D)** sema4d induced cell migration and growth-cone collapse.
